# Effect of hydrothermal aging temperature on a Cu-SSZ-13/H-SAPO-34 composite for the selective catalytic reduction of NO_*x*_ by NH_3_†

**DOI:** 10.1039/d1ra05168g

**Published:** 2021-10-11

**Authors:** Huawang Zhao, Mimi Lin, Yujie Wang, Jiandong Zheng

**Affiliations:** Department of Environmental Science & Engineering, College of Chemical Engineering, Huaqiao University Xiamen Fujian 361021 China hwzhao@hqu.edu.cn; Xiamen Power Supply Company, State Grid Fujian Electric Power Co. Ltd. Xiamen Fujian 361001 China; School of Materials and Chemical Engineering, Chuzhou University Chuzhou Anhui 239000 China

## Abstract

Cu-SSZ-13 suffers activity loss after hydrothermal treatment at high temperatures, particularly above 850 °C. The stability of Cu-SSZ-13 can be enhanced by compositing with H-SAPO-34. This work investigates the effect of aging temperature on the composites. For the structure, the extra-framework P in H-SAPO-34 migrates and interacts with the Al in Cu-SSZ-13, forming a new framework P–Al bond. This interaction is enhanced with the increment of the aging temperature. For the cupric sites, the aging at 750 °C results in the agglomeration of Cu^2+^ ions to CuO. However, the sample aged at 800 °C exhibits higher activities than that aged at 750 °C, which might be attributed to the increased formation of framework P–Al bonds promoting the redispersion of CuO to Cu^2+^ ions. The composite suffers severe deactivation due to the significant loss of Cu^2+^ ions after aging at 850 °C.

## Abstract 2

Effect of aging temperature on SSZ-SAPO composites.

## Graphical abstract

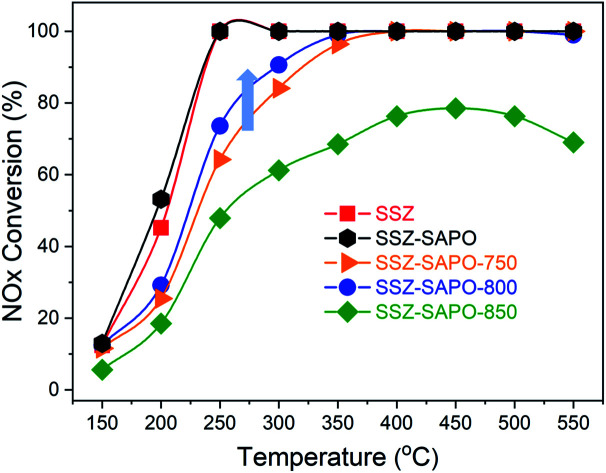

## 1 Introduction

Copper-exchanged zeolites, used in diesel vehicle after-treatment systems for selective catalytic reduction of NO_*x*_ with ammonia (NH_3_-SCR), have been investigated for decades.^1–4^ Cu/ZSM-5, Cu/beta, and Cu-chabazite, including Cu-SSZ-13 and Cu-SAPO-34, have been developed.^3^ Compared to other types of Cu-zeolite catalysts, Cu-SSZ-13 and Cu-SAPO-34 exhibit higher SCR activity and enhanced hydrothermal stability.^5,6^ Those advantages make it possible to commercialize the catalyst.

Both Cu-SSZ-13 and Cu-SAPO-34 adopt a small pore chabazite zeolite structure, and the isolated Cu^2+^ ion sites are identified as the NH_3_-SCR active sites.^3,7^ However, their responses to the hydrothermal aging (HTA) treatment are different. Typically, the Cu-SSZ-13 retains a high deNO_*x*_ activity after HTA at temperatures below 800 °C.^5,8–10^ However, with the HTA temperature increasing to 850 °C, the Cu-SSZ-13 suffers severe deactivation.^5^ The dealumination and agglomeration of Cu^2+^ ions to CuO_*x*_ are considered as two main contributions to the activity loss for Cu-SSZ-13 after HTA.^11^ Doping with promoters, such as Ce^4+^, Na^+^, K^+^, *etc.*, was reported to improve the HTA stability of Cu-SSZ-13.^12,13^ Precise adjustment of the framework Al position and distribution by changing the SSZ-13 synthesis recipe is also a rational approach.^8^

Unlike aluminosilicate SSZ-13, the SAPO-34 contains Si, Al, and P in the framework. The Cu-SAPO-34 shows a robust HTA stability when aged at high temperatures, which maintains a high SCR activity even after HTA at 850 °C.^5^ P in SAPO-34 has been proposed critical to stabilize the CHA type framework in hydrothermal condition.^14^

Cu-zeolites with a combination of two zeolite structures showed enhanced HTA stability over single catalyst. For example, Cu-ZST-1 (AFX/CHA),^15^ Cu-SAPO-34/5 (CHA/AFI),^16^ and Cu-OFF/CHA^17^ composites exhibited improved hydrothermal stability than the individual component. Our previous work also showed that the mixed Cu-SSZ-13/Cu-SAPO-34 composites performed a much better HTA stability than Cu-SSZ-13, due to the intense interaction between the two components (SSZ-13 and SAPO-34) during HTA.^18^ The P might promote the stability of the Cu-SSZ-13/Cu-SAPO-34 composite,^18^ and also, some works found that the P had negative effect on the stability of Cu-SSZ-13.^19–21^ The different P source, P doping content, Si/Al ratio and Cu loading content of Cu-SSZ-13, and HTA temperature applied in the works might contribute to the differences.^22–24^ However, the investigation on HTA effect on Cu-SSZ-13/Cu-SAPO-34 composites was only carried out at 750 °C in our previous work.^18^ As mentioned above, the HTA temperature imposes a big influence on the stability of Cu-SSZ-13, which might also affect the interaction between Cu-SSZ-13 and Cu-SAPO-34 component.^5^ This work aims to probe the changes of composites and to find the underlying interaction chemistry between the two components after HTA at different temperatures.

## 2 Experimental

### 2.1 Catalysts preparation

The Na-SSZ-13 was synthesized using the procedure reported in our previous works.^8,25^ Firstly, the calcined Na-SSZ-13 was transformed into NH_4_-SSZ-13 *via* ion-exchange with a 5 M (NH_4_)_2_SO_4_ solution at 80 °C for 2.5 h. Subsequently, 2 g of NH_4_-SSZ-13 was dispersed in 200 mL 0.008 M CuSO_4_ solution. The mixture was heated to 80 °C and kept for 1 h under stirring. After each step, the samples were collected *via* filtration, washed with deionized water, and dried overnight at 110 °C. Finally, the Cu exchanged samples were calcined in static air at 600 °C for 6 h with a ramp of 5 °C min^−1^, obtaining the Cu-SSZ-13 sample. The Si/Al ratio and Cu content in Cu-SSZ-13 were determined by ICP-OES (VISTAMPX, Varian), which were 6 and 2.38 wt%, respectively. The Cu/Al ratio of Cu-SSZ-13 is around 0.22. The H-SAPO-34 (chemical composition: P_2_O_5_ : SiO_2_ : Al_2_O_3_ = 1 : 1 : 1) was purchased from Nankai University catalysts Co., Ltd.

Our previous work has evaluated the hydrothermal stability of Cu-SSZ-13/Cu-SAPO-34 composites with different weight ratio of two components (Cu-SSZ-13 : Cu-SAPO-34 = 9 : 1, 7 : 3, 5 : 5, respectively), and the composite with the weight ratio of 5 : 5 exhibited the most robust stability.^18^ This work focusses on investigating the effect of hydrothermal aging temperature on the composite. Therefore, the optimized Cu-SSZ-13/H-SAPO-34 with weight ratio of 5 : 5 was used. The fresh Cu-SSZ-13 and H-SAPO-34 powders were mixed mechanically with a mortar, and the weight ratio of the two component is 5 : 5. The Cu-SSZ-13 and composites were hydrothermally treated in 10 vol% H_2_O/air at 750, 800, and 850 °C for 16 h, respectively. The flowrate of feed in HTA is 500 mL min^−1^. The mixed Cu-SSZ-13 and H-SAPO-34 composites is denoted as SSZ-SAPO. The hydrothermal aging (HTA) composites are abbreviated as SSZ-SAPO-*X*, where *X* represents the aging temperature.

### 2.2 Activity measurement

The SCR activity was measured in a fixed-bed quartz reactor with the diameter of 6 mm. The feed gases contain 500 ppm NO, 500 ppm NH_3_, 5% O_2_, 5% H_2_O, and N_2_ as balance. 50 mg of the Cu-SSZ-13 (40–60 mesh) diluted by 300 mg of quartz sands was loaded in the reactor. To better compare the performance of Cu-SSZ-13 and SSZ-SAPO samples, 100 mg of the SSZ-SAPO composites (40–60 mesh) diluted by 250 mg of quartz sands was used for the activity tests. As such, the Cu content in Cu-SSZ-13 and SSZ-SAPO composites for tests is the same. The catalysts were activated at 600 °C in 5% O_2_/N_2_ flow for 1 h prior to the NH_3_-SCR tests. The reaction temperature was increased from 150 °C to 550 °C. The effluent gas composition was analyzed online with a Testo 350 Gas Analyzer. The NO_*x*_ (NO_*x*_ = NO + NO_2_) conversion was calculated using the following equation:1
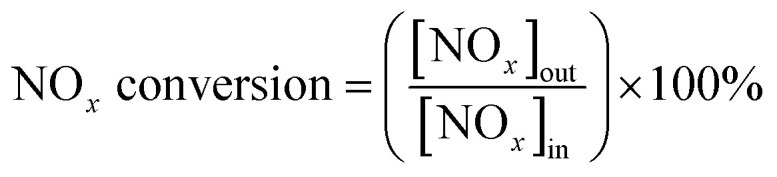


### 2.3 Characterization

The powder X-ray diffraction (XRD) pattern was recorded with a Bruker AXS instrument with a Cu Kα radiation. The scan angle extended from 5° to 50° with a scan rate of 2° min^−1^.

^27^Al and ^31^P solid-state Magic Angle Spinning Nuclear Magnetic Resonance (MAS NMR) were conducted on Varian Infinity plus 300 WB spectrometer utilizing a 4 mm triple resonance probe operating with the resonance frequencies of 78.13 MHz and 40 kHz, respectively. Spectra of ^27^Al were acquired by using calibrated ^27^Al π/20 pulses of 0.5 μs, a 40 kHz spectral window, a spinning speed of 8 kHz, and a 3 s pulse delay. Al(NO_3_)_3_ aqueous solution (1 mol L^−1^) was used for ^27^Al MAS NMR spectroscopy as reference. ^31^P MAS NMR was conducted with a spinning speed of 9 kHz, and a 15 s pulse delay. H_3_PO_4_ aqueous solution (75 wt%) was used for ^31^P MAS NMR spectroscopy as a reference.

Electron paramagnetic resonance (EPR) spectra in the X-band were recorded with a CW spectrometer JES-FA200, with a microwave power of 1 mW modulation frequency of 100 kHz. The EPR signals of isolated Cu^2+^ ions were recorded at −150 °C with the magnetic field being swept from 2000 to 4000 G with a sweep time of 5 min.

*In situ* Diffuse Reflectance Infrared Fourier Transform Spectroscopy (DRIFTs) was performed using a Bruker Vertex 70 spectrometer equipped with an MCT detector. The catalyst was first pretreated in 10% O_2_/N_2_ for 60 min at 600 °C and cooled to 25 °C in the same atmosphere. The background was collected after the sample stabilizing at 25 °C for 10 min. The IR spectra were collected after the NH_3_ adsorption for 30 min and then purged by N_2_ for 5 min.

Temperature-programmed reduction by H_2_ (H_2_-TPR) measurements were performed on an AutoChem 2920 apparatus with a TCD detector. 75 mg of sample was heated to 600 °C in the 5% O_2_/N_2_ before the test. After cooling down to room temperature, the sample was exposed to 5% H_2_/N_2_, with the temperature increasing to 900 °C at a rate of 10 °C min^−1^, where the flow rate was 30 mL min^−1^.

Scanning electron microscopy (SEM) images were obtained on a SEM s4800 microscope (Hitachi) operated at 10 kV. The powders were placed on a sample holder with carbon tape and covered with Au film to improve the conductivity. Energy dispersive spectroscopy (EDS) mapping was also performed in the same instrument operated at 10 kV.

## 3 Results

### 3.1 Catalytic performance

**Fig. 1:**
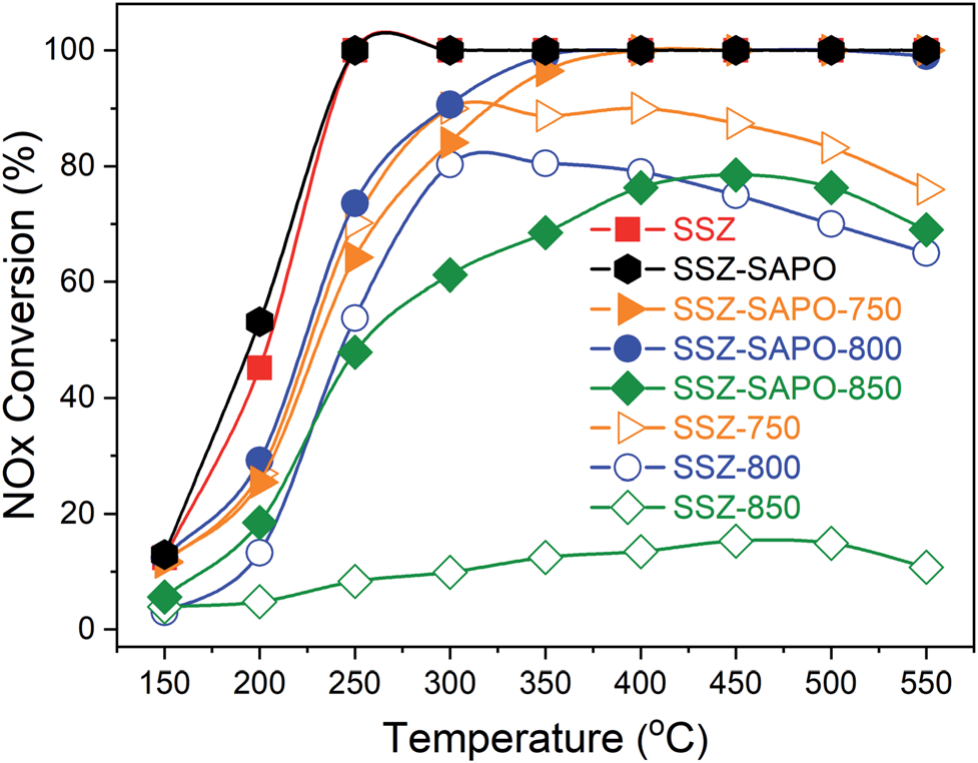
NO_*x*_ conversion of fresh and HTA samples during standard NH_3_-SCR as a function of the temperature.

The activity of Cu-SSZ-13, fresh and aged SSZ-SAPO mixed samples are shown in Fig. 1. The Cu-SSZ-13 and SSZ-SAPO samples exhibited similar deNO_*x*_ activities, showing the NO_*x*_ conversion of around 50% at 200 °C and keeping 100% conversion in 250–550 °C. After HTA, the NO_*x*_ conversion of Cu-SSZ-13 decreased with the increase of the HTA temperature. Particularly, the Cu-SSZ-13 was completely deactivated after HTA at 850 °C, with the maximum NO_*x*_ conversion of 18% at around 400 °C.

The SSZ-SAPO composite showed an activity decline after HTA at 750 °C, showing the NO_*x*_ conversion of 63% and 82% at 250 °C and 300 °C, respectively, while achieving 100% conversion above 400 °C. Interestingly, the SCR performance of the composite was improved after HTA at 800 °C, where the NO_*x*_ conversion at 250 °C and 300 °C was 73% and 90%, respectively, around 10% higher than that observed in SSZ-SAPO-750. With the HTA temperature increasing to 850 °C, the SSZ-SAPO still presented a high deNO_*x*_ activity with the maximum conversion of 72% at 450 °C.

Generally, compared to Cu-SSZ-13, the composites exhibited much higher activities after HTA, indicating the higher stability of the composite than Cu-SSZ-13, consistent with our previous report.^18^ It indicates that the synergistic effect occurred between Cu-SSZ-13 and H-SAPO-34 during HTA, which would be discussed in Section 4.1. Besides, the better SCR performance of SSZ-SAPO-800 than SSZ-SAPO-750 suggests that the synergistic effect is highly dependent on the HTA temperature.

### 3.2 Structural characterizations with XRD, ^27^Al MAS NMR, and ^31^P MAS NMR

**Fig. 2:**
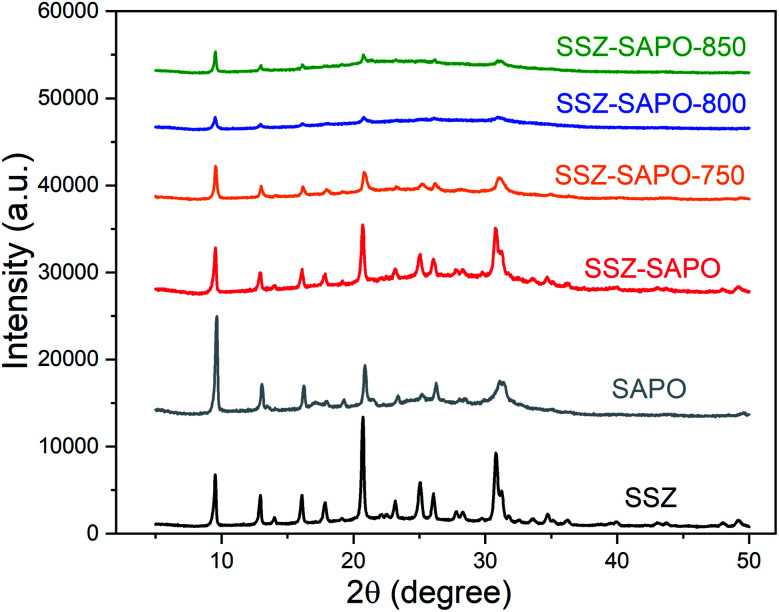
XRD patterns of the fresh and the aged catalyst samples. The relative crystallinity is given above each pattern.

The Cu-SSZ-13, H-SAPO-34, and SSZ-SAPO composite displayed typical CHA structure (2*θ* = 9.5, 14.0, 16.1, 17.8, 20.7, and 25.0°) in Fig. 2, indicating the physical mixture did not change the crystalline of Cu-SSZ-13 and H-SAPO-34. The diffraction CHA peaks were remained in the composites after HTA, while the intensities of peaks decreased, suggesting the decrease of crystallinity. It should be pointed that the intensity of diffraction peak at around 9.5° is higher in SSZ-SAPO-850 than SSZ-SAPO-800, as shown in Fig. 2. The peak position also shifted to lower angle in SSZ-SAPO-850. According to the previous work,^26^ the peak at 9.5° represents the (100) plane in CHA crystalline. The higher intensity and lower angle of peak at 9.5° in SSZ-SAPO-850 than SSZ-SAPO-800 indicates the changes of crystallinity of the composites upon HTA at different temperatures.

**Fig. 3:**
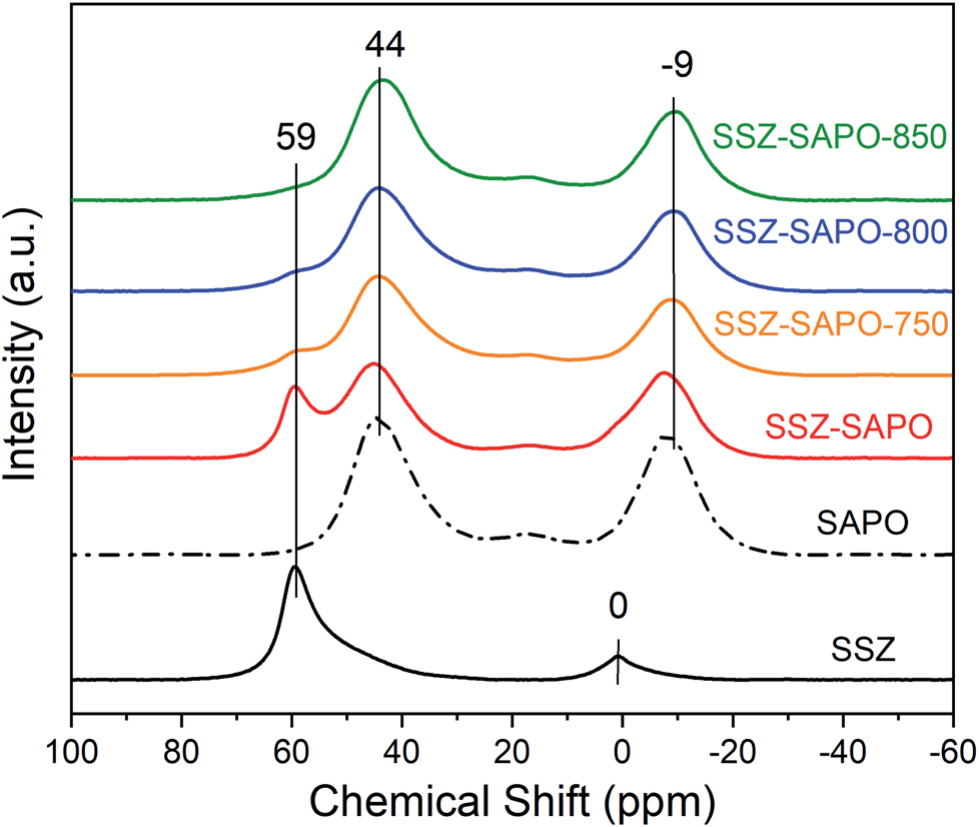
Solid state ^27^Al MAS NMR spectra of the fresh catalysts and HTA catalysts.

The dealumination during HTA would result in the crystalline decomposition for SSZ-13 and SAPO-34.^14,27–29^ The ^27^Al MAS NMR technique is sensitive to the subtle changes of framework Al in Cu-SSZ-13 and H-SAPO-34. To monitor the changes in chemical environment of Al in the composites after HTA, the ^27^Al MAS NMR measurements were carried out. The results are shown in Fig. 3.

Cu-SSZ-13 displayed a dominate resonance peak at 59 ppm and a small peak at around 0 ppm, which is attributed to the tetrahedral framework aluminum (TFAl) and octahedral extra-framework aluminum, respectively.^29,30^ The peaks at −9 and 44 ppm were observed in H-SAPO-34, which is assigned to octahedrally coordinated Al(OP)_4_(H_2_O)_2_ and Al(OSi)(OP)_3_(H_2_O)_2_ and tetrahedrally coordinated (Al(OP)_4_ and Al(OSi)(OP)_3_) framework Al atoms, respectively.^18^ Both the octahedrally and tetrahedrally coordinated Al in H-SAPO-34 belong to the framework Al.^18^ In the SSZ-SAPO composite, the overlapping peaks of fresh Cu-SSZ-13 and H-SAPO-34 were observed.

The resonance peaks changed significantly after HTA. The TFAl peak at 59 ppm in the composites decreased after HTA at 750 and 800 °C, which disappeared in SSZ-SAPO-850. In contrast, the TFAl coordinated to P (−10–40 ppm) increased with the increment of HTA temperature. It indicates that some new framework Al species were generated in the composite during HTA, where the phosphorous should play a key role.

**Fig. 4:**
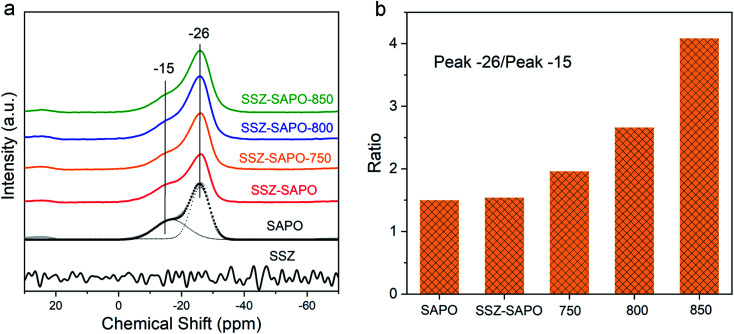
(a) Solid state ^31^P MAS NMR spectra of the fresh catalysts and HTA catalysts. (b) The area ratio of peak at −26 to peak −15.

To determine the changes of phosphorous species in the mixture after HTA, ^31^P MAS NMR measurement was conducted. As shown in Fig. 4a, two peaks at −26 and −15 ppm were observed in H-SAPO-34, attributed to the tetrahedrally coordinated framework P (P(OAl)_4_) and extra-framework P (P(OAl)_*x*_(H_2_O)_*y*_), respectively.^26^ The two resonance peaks remained in the composite after HTA. However, the intensity of the extra-framework P (−15 ppm) decreased with the increment of the HTA temperature, while the framework P at −26 ppm increased. Quantitatively, the peak area ratio of the peaks at −26 and −15 is given in Fig. 4b, the ratio value increases with the increasing HTA temperature, indicating that the of P(OAl)_4_ increased and the of P(OAl)_*x*_(H_2_O)_*y*_ decreased. This change can be explained by the transformation of extra-framework P to the framework P during HTA. Conjunction with the ^27^Al MAS NMR (Fig. 3) and ^31^P MAS NMR results, it is convincing to infer that some new framework P–Al species were formed in the composite during HTA, and the higher temperature increased the formation of new framework P–Al.

### 3.3 Characterizations of the cupric sites

#### 3.3.1 EPR results

**Fig. 5:**
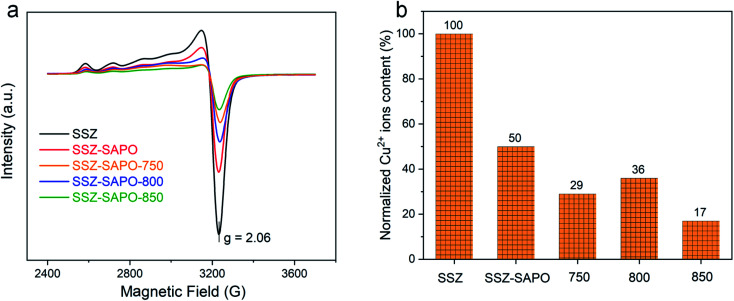
Electron paramagnetic resonance (EPR) spectra (a) and the normalized Cu^2+^ ions concentration (b) of the catalyst samples. The normalized Cu^2+^ ions concentration was determined with double integrating the EPR spectra, using the isolated Cu^2+^ ions content in Cu-SSZ-13 as the reference. Spectra were collected at −150 °C.

The isolated Cu^2+^ ions are identified as the active sites for NH_3_-SCR reaction.^1,31^ The changes of deNO_*x*_ activity of the composites after HTA (Fig. 1) should be highly related to the variation of the Cu^2+^ ions. The EPR measurements were firstly carried out to describe the changes of Cu^2+^ ions after HTA, since Cu^2+^ ions are EPR active and other cupric species such as CuO are inactive.^31^ The results are shown in Fig. 5a.

All samples showed the same EPR line-shape with a *g* value of 2.06, indicating the same Cu^2+^ ions state. It should be pointed out that the amount of sample used in each EPR measurement is the same. Therefore, the Cu^2+^ ions content in fresh and HTA samples can be normalized by double integrating the EPR curves, using Cu-SSZ-13 as a reference. The results are shown in Fig. 5b. As H-SAPO-34 does not give EPR signal, the Cu^2+^ ions content of SSZ-SAPO is half that of the Cu-SSZ-13. 29% of Cu^2+^ ions remained in the SSZ-SAPO-HTA-750, which increased to 36% after HTA at 800 °C. After HTA at 850 °C, the Cu^2+^ ions content decreased to 17%. It indicates that the HTA temperature has a big influence on the Cu^2+^ ions content in the composite.

#### 3.3.2 NH_3_-DRIFTs

**Fig. 6:**
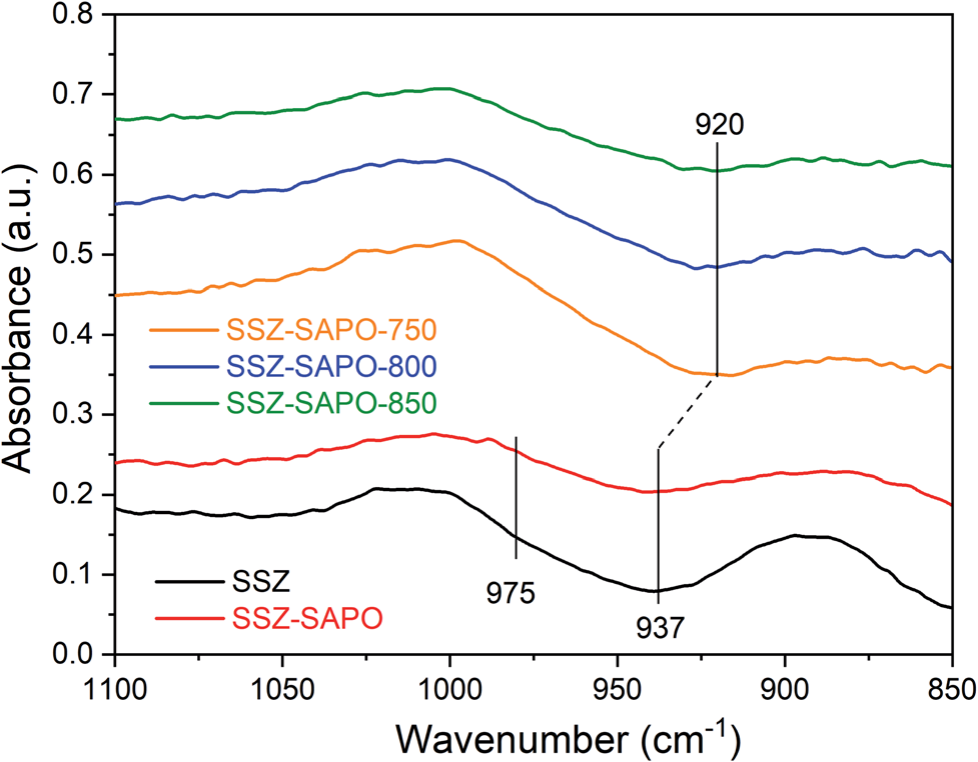
NH_3_-DRIFTS spectra after NH_3_ adsorption on fresh and HTA samples. Conditions: 500 ppm NH_3_/N_2_ at 25 °C, then N_2_ purge before spectra collection.

It has been widely reported that two kinds of Cu^2+^ cations, *i.e.*, [Cu(OH)]^+^ and Cu^2+^-2Al, exist in Cu-SSZ-13.^32,33^ The two types of Cu^2+^ ions display different negative framework vibrations bands in the NH_3_-saturated Cu-SSZ-13, when using a dehydrated Cu-SSZ-13 as the background for IR analysis.^34,35^ Therefore, NH_3_-DRIFTs technique was used to gain further insights into hydrothermal aging effects on the cupric sites. The results are shown in Fig. 6.

For the fresh Cu-SSZ-13, a dominant negative T–O–T band at 937 cm^−1^ and a small shoulder peak at 975 cm^−1^ were observed, assigned to the Cu^2+^ coordinated to paired Al sites in six-member rings (Cu^2+^-2Al) and Cu^2+^ ions balanced by a single framework Al in eight-member ring (Cu(OH)^+^), respectively.^11^ The positions of the two negative bands remained unchanged in the fresh SSZ-SAPO composite.

After HTA, the negative band at 975 cm^−1^ disappeared, and the band at 937 cm^−1^ band shifted to ∼920 cm^−1^ but remained a high intensity. It indicates that a large content of Cu^2+^-2Al were still maintained in the HTA samples. Besides, the redshift of the IR band suggests that the interaction between Cu^2+^ ions and the framework became weaker after HTA.^36^

#### 3.3.3 H_2_-TPR results

**Fig. 7:**
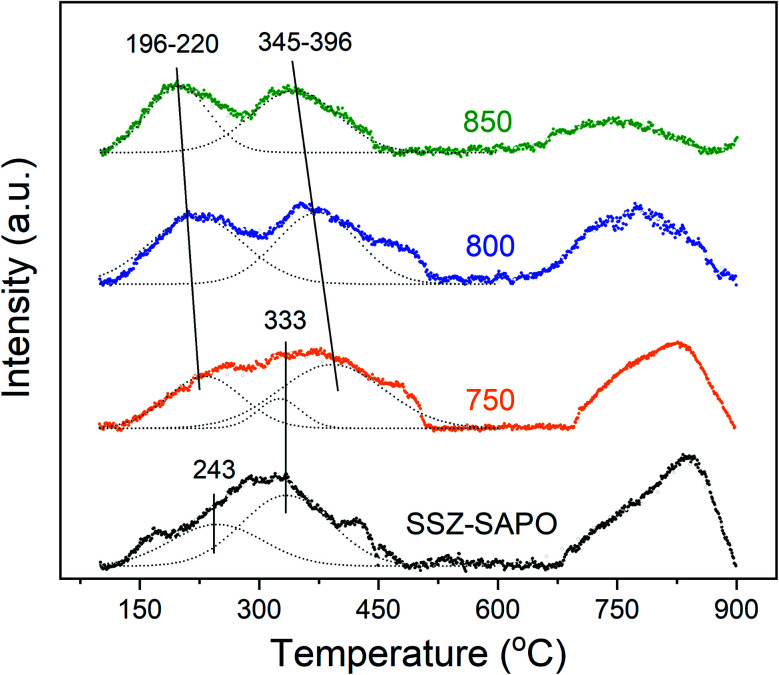
H_2_-TPR spectra of the catalyst samples.

To acquire more information of the state of cupric sites in the fresh and HTA samples, the H_2_-TPR measurement were carried out. The results are shown in Fig. 7. The EPR (Fig. 5) and NH_3_-DIRFTs (Fig. 6) results showed that the H-SAPO-34 has no influence on the cupric species in Cu-SSZ-13. Thus, the H_2_-consumption peak should be exclusively due to the reduction of cupric sites in Cu-SSZ-13 component. Indeed, the line-shape of the H_2_-TPR curves of Cu-SSZ-13 and SSZ-SAPO composite are the same (Fig. S6†). To simplify the analysis of the H_2_-TPR results, the reduction profile of Cu-SSZ-13 is not given in the main text.

Two distinct reduction ranges were found in the SSZ-SAPO sample: one above and one below 500 °C. The reduction peak below 500 °C can be attributed to the reduction of Cu^2+^ to Cu^+^ (Cu^2+^ + ½H_2_ = Cu^+^ + ½H_2_O) or the reduction of CuO to Cu^0^ (CuO + ½H_2_ = Cu^0^ + ½H_2_O).^37^ The integral peak area below and above 500 °C is same in SSZ-SAPO (Table S1†), indicating that the cupric species in Cu-SSZ-13 are exclusively Cu^2+^ ions. The reduction peak below 500 °C can be deconvoluted into two peaks centered at 243 and 333 °C (with a shoulder peak at 430 °C), which is assigned to [Cu(OH)]^+^ and Cu^2+^-2Al, respectively, as the Cu^2+^-2Al is more stable than Cu(OH)^+^.^32,38^ The much higher peak area of the peak at 350 °C (Table S1†) indicates that the Cu^2+^-2Al ions are dominate in the Cu-SSZ-13, consistent with the NH_3_-DRIFTs results in Fig. 6.

The peak below 500 °C was broadened after HTA at 750 °C, which can be deconvoluted into three peaks, at 220, 333, and 396 °C (with a shoulder peak at 470 °C in SSZ-SAPO-750), respectively. The peak at 333 °C should be attributed to the reduction of Cu^2+^-2Al, as discussed above. Moreover, the NH_3_-DRIFTs results presented that a new type of Cu^2+^ ions (named as Cu^2+^-new) were formed in the HTA-750 sample. In addition, a part of Cu^2+^ ions were transformed to CuO after HTA, based on the EPR and TEM (Fig. S7†) results. Therefore, the peak at 200 °C and 396 °C (with a shoulder peak at 470 °C in SSZ-SAPO-800) should be related to the reduction of Cu^2+^-new ions and CuO, respectively. Since the Cu^2+^-new ions have a low binding energy with the framework (Fig. 6), the reduction temperature of which should be lower than Cu^2+^-2Al. Therefore, the peak at 220 °C is attributed to the reduction of the Cu^2+^-new ions, and the peak at 396 °C is assigned to the CuO.

Also, two distinct reduction states were found in the SSZ-SAPO-800 and SSZ-SAPO-850 samples at temperatures below and above 500 °C. After deconvolution, only two peaks centered at around 200 and 370 °C were found, assigned to the Cu^2+^-new ions and CuO, respectively. The formation of CuO particles can be confirmed by the TEM images of SSZ-SAPO-850 in Fig. S8.† The reduction temperature of Cu^2+^-new ions and CuO decreased with the increment of HTA temperature, which should be related to the changes of the framework.^29^ This part will be discussed in Section 4.2.

### 3.4 The redispersion of P, Al, and Cu characterized by EDS mapping

**Fig. 8:**
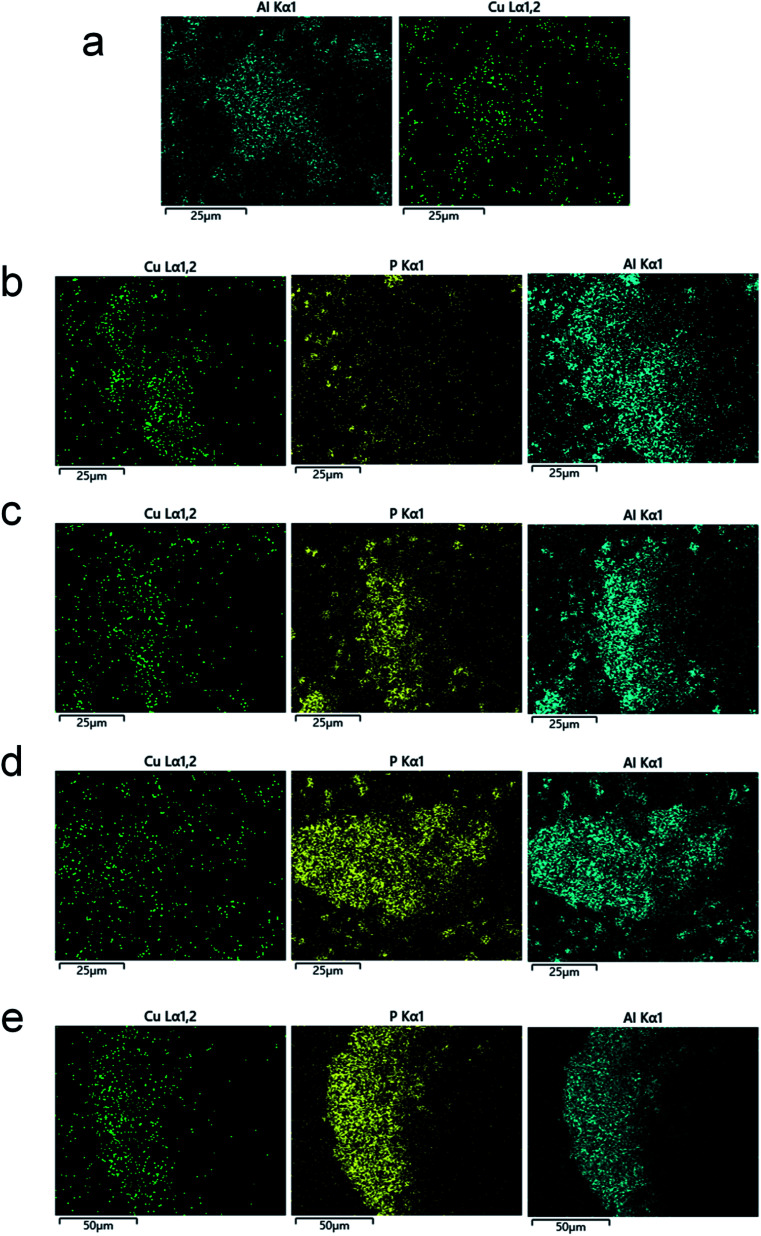
EDS mapping of the catalyst samples. The (a, b, c, d, and e) refer to Cu-SSZ-13, SSZ-SAPO, SSZ-SAPO-750, SSZ-SAPO-800, and SSZ-SAPO-850, respectively.

The ^27^Al MAS NMR (Fig. 3), ^31^P MAS NMR (Fig. 4), NH_3_-DRIFTs (Fig. 6), and H_2_-TPR (Fig. 7) results showed that the framework P, TFAl, and Cu^2+^ ions were changed after HTA. This variation can be visible through the EDS mapping. The results are shown in Fig. 8. It should be pointed out that the diameter of Cu-SSZ-13 and H-SAPO-34 is lower than 1 μm, while the scale of images is larger than 100 × 100 μm. Therefore, the mapping images can represent the overall dispersion of the P, Al, and Cu in the samples.

In the Cu-SSZ-13, highly dispersed Si, Al and Cu species were observed. The Cu dispersion was highly consistent with the Al dispersion, because the framework Al in Cu-SSZ-13 provided the ion-exchange sites for Cu^2+^ ions.^1^ In the fresh SSZ-SAPO, in addition to the Cu, Al, and Si, the highly dispersed P atoms were also found. As P atoms are only contained in H-SAPO-34 component in the mixed sample, the highly dispersed P atoms demonstrates that the H-SAPO-34 and Cu-SSZ-13 were thoroughly mixed in the SSZ-SAPO composite. Besides, the distribution of P and Cu was inconsistent in the mixed sample, suggesting that the Cu^2+^ ions were only contained in Cu-SSZ-13 component.

The coincidence extent of P and Al distribution increased with the increasing HTA temperature, as compared Fig. 8a–e. Particularly, the P and Al distribution were fully consistent in the SSZ-SAPO-850 sample. This suggests that the P and Al in the two Cu-SSZ-13 and H-SAPO-34 components tended to migrate close to each other during HTA. In contrast, the distribution of Cu and Al becomes inconsistent after HTA. This can be explained by the formation of CuO particles, which did not occupy the ion-exchange sites.

## 4 Discussion

### 4.1 The evaluation of the framework after HTA at different temperatures

The formation of a new framework P–Al bond should be related to the redispersion of P and Al in SSZ-SAPO composites during HTA (Fig. 3, 4, and 8). According to the previous report,^1^ a healing process, *i.e.* the migration of P atoms to framework vacancies transforming to P(OAl)_4_ species, was believed to contribute to the ultra-high stability of the framework of SAPO-34. Also, in our previous works,^14,18^ the migration of P to heal the Cu-SSZ-13 framework during HTA were also observed, using the HPO_3_ and Cu-SAPO-34 as the P source, respectively. In this case, the extra-framework P in SSZ-SAPO composite decreased (Fig. 4), while the framework P–Al bands increased after HTA (Fig. 3 and 4), indicating that the healing process by extra-framework P occurred. Besides, there was no extra-framework Al existing in H-SAPO-34 component (Fig. 3). This demonstrates that the formation of new framework P–Al bands should be attributed to the migration of extra-framework P atoms in SAPO component to interact with the Al from SSZ component during HTA.

The interaction of P and Al in the composite was enhanced with the increasing HTA temperature from 750 to 850 °C, as shown in ^27^Al and ^31^P MAS NMR results (Fig. 3 and 4). Particularly, the highly consistent distribution of P and Al as well as the compete disappearance of TFAl in SSZ-SAPO-850 (Fig. 8) indicates that the two individual Cu-SSZ-13 and H-SAPO-34 components in fresh SSZ-SAPO sample transformed into a pure silicoaluminophosphate substance. This might be attributed to the enhanced migration ability of P atoms in SAPO-34 at higher HTA temperatures, facilitating the interaction between P and Al.

### 4.2 The evaluation of the cupric sites after HTA at different temperatures

The NH_3_-DRIFTs (Fig. 6) and H_2_-TPR (Fig. 7) results showed that the bonding energy between the reaming Cu^2+^ ions and the framework became weaker in the SSZ-SAPO after HTA, particularly for the SSZ-SAPO-800 and SSZ-SAPO-850 samples. According to the previous works,^33^ the boding energy between Cu^2+^ ions and framework Al was weaker in Cu-SAPO-34 than Cu-SSZ-13, which might be due to the framework P reduce the electronegativity of the skeleton. Therefore, the reduced reduction temperature of the Cu^2+^ ions in HTA SSZ-SAPO composites (Fig. 7) can be rationalized by the formation of new framework P–Al bond in the SSZ-13 component during HTA. Alone this line, the increased framework P–Al bond in the SSZ-SAPO composites which aged at higher temperatures resulted in the lower reduction temperature of Cu^2+^ ions (Fig. 6 and 7).

The Cu^2+^ ions content is higher in SSZ-SAPO-800 than SSZ-SAPO-750 (Fig. 5), suggesting that less CuO was formed after HTA at 800 °C. This can be confirmed by the smaller area of the CuO reduction peak in SSZ-SAPO-800 than SSZ-SAPO-750, according to the H_2_-TRP results in Table S1.† However, it is a well-known notion that Cu^2+^ ions in Cu-SSZ-13 would suffer a more severe agglomeration after HTA at higher temperatures.^5,39,40^ This contradiction demonstrates that SAPO-34 component contributed to the improved activity of SSZ-SAPO-800. It was reported that CuO would migrate from the external surface into the micropores of SAPO-34 during the hydrothermal treatment and form isolated Cu ions at the exchange sites, which led to the increase of Cu^2+^ ions.^41,42^ The pathway redispersion of CuO to Cu^2+^ might be attributed to the *via* protonolysis of CuO particles on Brønsted sites (eqn (2) and (3)).^41^

2 2CuO + Z-H + H_2_O = 2Z-Cu(OH)^+^

3 CuO + 2Z-H = Z_2_-Cu^2+^ + H_2_O

Z represents the framework (Si–O–Al)^−^ and Z_2_-Cu^2+^ represents the Cu^2+^-2Al

However, this phenomenon was not observed in Cu-SSZ-13.^29^ Apparently, the increased framework silicoaluminophosphate interface in SSZ-SAPO-800 than SSZ-SAPO-750 (Fig. 3 and 8) would facilitate the transformation of CuO to Cu^2+^, resulting in a higher Cu^2+^ ions content (Fig. 5b) and a better SCR performance of SSZ-SAPO-800 sample (Fig. 1).

Compared to the SSZ-SAPO-800 which still contained the SSZ-13 competent, the SSZ-SAPO-850 became a pure silicoaluminophosphate material (Fig. 3 and 8). However, SSZ-SAPO-850 contained a much lower content of Cu^2+^ ions than that SSZ-SAPO-800 (Fig. 5). It is known that, in addition to the redispersion of CuO to Cu^2+^ ions, the migration and collision of the hydrolyzed Cu^2+^ ions to CuO also occurred in Cu-SAPO-34 if aged at high aging temperatures (850 °C and above),^5,27^ which follows the pathway below:

4 Z_2_-Cu^2+^ + H_2_O = Cu(OH)_2_ + 2Z-H

5 Cu(OH)_2_ + Cu(OH)_2_ = 2CuO + 2H_2_O

This indicates that the transformation of different kinds of Cu following eqn (2)–(5) should be in a transit status, which is highly dependent on the HTA temperatures. In other words, CuO would be redispersed to CuO at 800 °C, while the pathway that Cu^2+^ ions agglomerated to CuO was dominated when HTA at 850 °C in SSZ-SAPO composite. This can probably be attributed to the higher mobility of Cu^2+^ ions at higher HTA temperature,^11^ which resulted in the more severe agglomeration of Cu^2+^ ions after HTA at 850 °C (Fig. 5 and 7), leading to the inferior SCR activity of the SSZ-SAPO-850 than that of SSZ-SAPO-800 composites (Fig. 1).

## 5 Conclusion

The effect of HTA temperature on Cu-SSZ-13/H-SAPO-34 composites was investigated. The results showed that the hydrothermal stability of the composite samples was significantly improved compared to that of the single Cu-SSZ-13, indicting the beneficial effect of H-SAPO-34 to the Cu-SSZ-13. The extra-framework P atoms in SAPO-34 was found migrating and interacting with the Al from SSZ-13 to form a new framework P–Al bond, and this process was enhanced with the increasing HTA temperature. After HTA at 850 °C, the Cu-SSZ-13/H-SAPO-34 composite became a pure silicoaluminophosphate substance.

For the cupric sites, the agglomeration of isolated Cu^2+^ ions to CuO resulted in the decline of activity after HTA at 750 °C. The HTA temperature to 800 °C increased the activity, compared to the composite aged at 750 °C, which might be due to the increment of framework P–Al bonds formation. While, after HTA at 850C, the composite suffered a severe deactivation, which can be attributed to the agglomeration of Cu^2+^ ions to CuO.

## Conflicts of interest

There are no conflicts to declare.

## Supplementary Material

RA-011-D1RA05168G-s001
