# Supplementary material for: Effect of hydrothermal aging temperature on a Cu-SSZ-13/H-SAPO-34 composite for the selective catalytic reduction of NOx by NH3
Source: RSC Adv. 2021 Oct 11;11(53):33334–43. doi: 10.1039/d1ra05168g (PMC9042250; doi:10.1039/d1ra05168g)
Supplement: RA-011-D1RA05168G-s001 [file RA-011-D1RA05168G-s001.pdf]

Electronic Supplementary Material (ESI) for RSC Advances.
This journal is © The Royal Society of Chemistry 2021

Supplenmtary Information

Effect of hydrothermal aging temperature on Cu-SSZ-13/H-SAPO-34 composite for the selective catalytic reduction of NOx by NH_3

Huawang Zhao [a*], *Mimi Lin* [b], *Yujie Wang* [c], *Jiandong Zheng* [c]

a. Department of Environmental Science & Engineering, College of Chemical Engineering, Huaqiao University, Xiamen, Fujian 361021, China

b. Xiamen Power Supply Company, State Grid Fujian Electric Power Co. Ltd., Xiamen, Fujian 361001, China

c. School of Materials and Chemical Engineering, Chuzhou University, Chuzhou, Anhui 239000, China

[*] Corresponding authors: Email: hwzhao@hqu.edu.cn

Table S1. Calculated areas of the area different divided peaks of the samples

Samples	243 °C [a]	333 °C [a]	396 °C [a]	Above 500 °C [a]
SSZ-SAPO	0.97	1.60		2.53
SSZ-SAPO-750	1.07	0.42	2.16	1.46
SSZ-SAPO-800	1.85		1.45	1.86
SSZ-SAPO-850	0.87		1.14	0.59

a. Obtained from Fig. 7. The peak was deconvoluted by Peakfit, and the values of area only represent the intensity.

The peaks at 243 and 333 °C represent the reduction of $Cu(OH)^+$ (also Cu^{2+}-new) and Cu^{2+}-2Al, respectively. The peak above 500 °C can be attributed to the reduction of Cu^+ to Cu^0. The details of the of the assignment of each reduction peak can be found in the main text (Section 3.3.3). The area values of the peak at 243 and 333 °C, representing the Cu^{2+} ions content in each sample, decreased after aging at 750 °C, and increased in the sample aged at 800 °C, and then decreased after aging at 850 °C, consistent with the trend of Cu^{2+} ions content changes in EPR results in Fig. 5.

The peak area of Cu^{2+} to Cu^+ in SSZ-SAPO-850 is higher than that of Cu^+ to Cu^0. This can be explained by that some highly stable Cu^+ ions are reduced at temperatures higher than 900 °C [1].

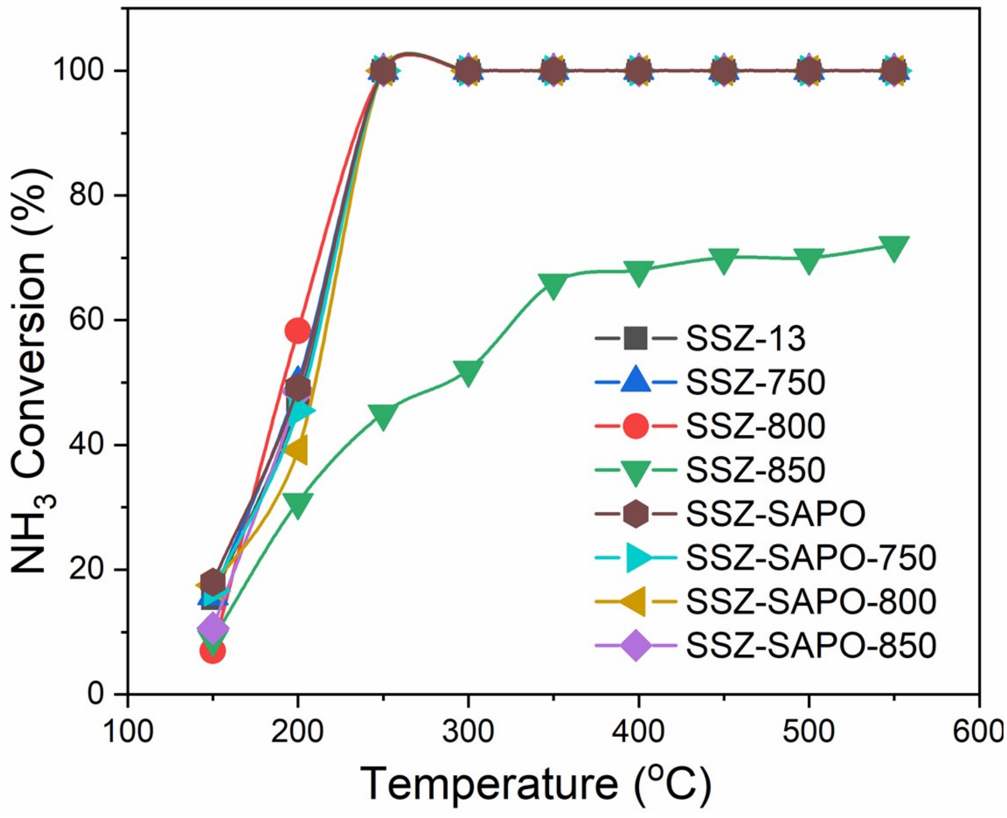

Fig. S1. NH_3 conversion as a function of temperature in NH_3-SCR reaction.

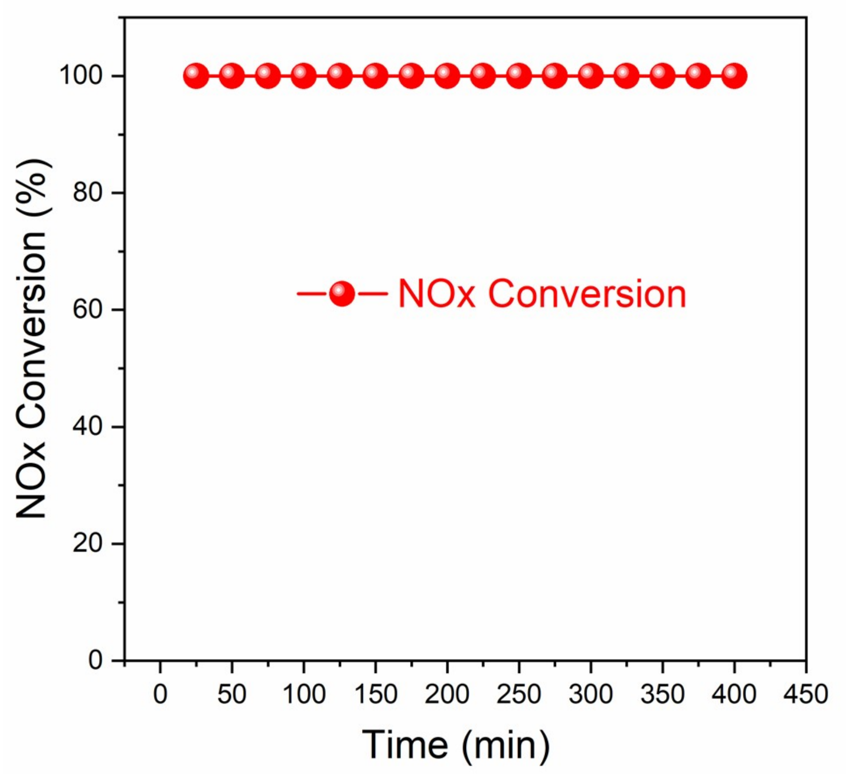

Fig. S2. Long-time NOx conversion of Cu-SSZ-13 at 250 °C.

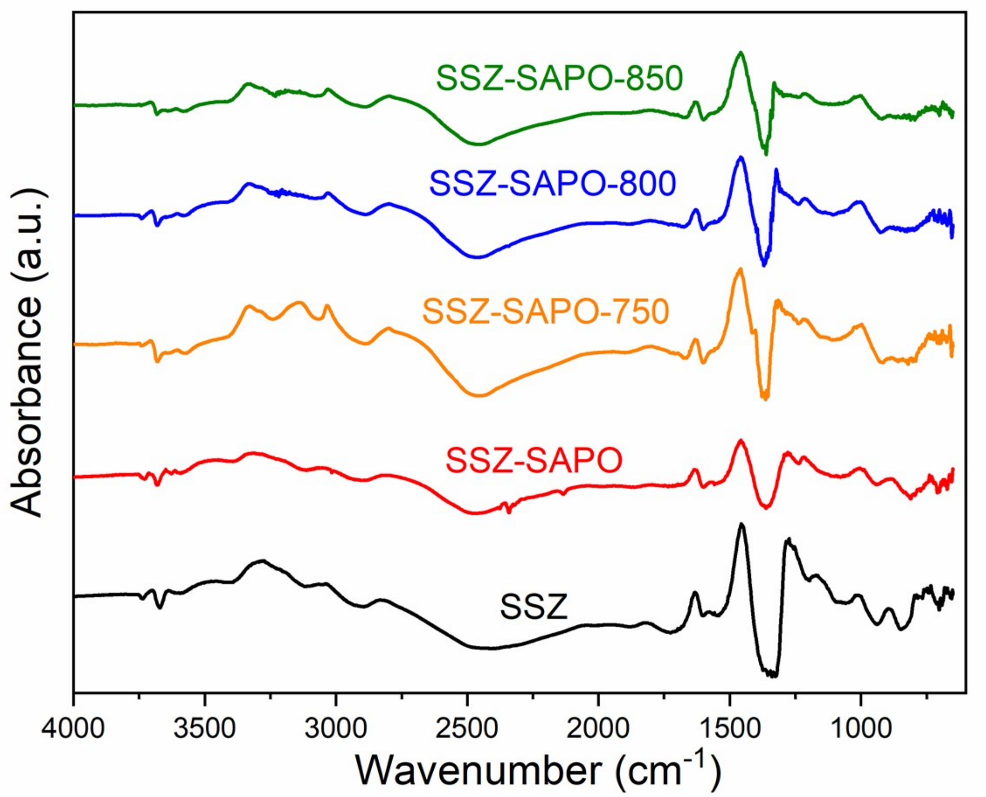

Fig. S3. DRIFTs spectra after NH_3 adsorption on the samples in 4000-500 cm^{-1} region. Conditions: 500 ppm NH_3/N_2 at 25 °C, then N_2 purge before spectra collection.

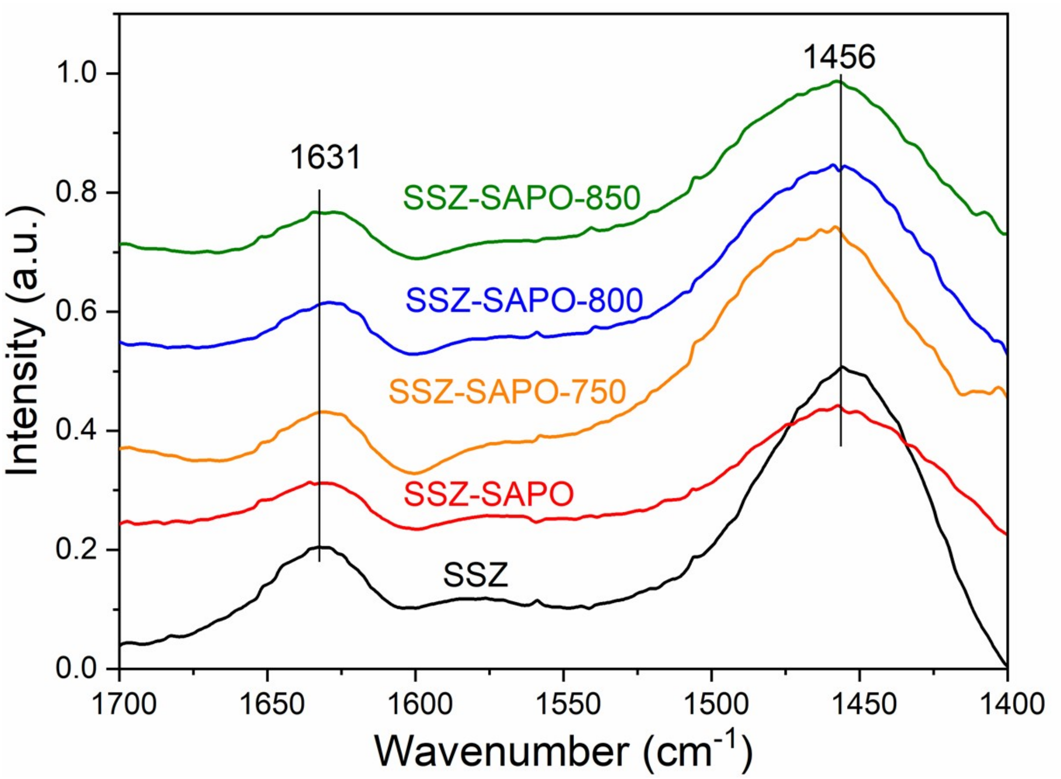

Fig. S4. DRIFTs spectra after NH_3 adsorption on the samples in 1700-1400 cm^{-1} region. Condition: 500 ppm NH_3/N_2 at 25 °C, then N_2 purge before spectra collection.

The bands at 1632 and 1475 cm^{-1} are attributed to molecular NH_3 absorbed onto exchanged Cu-related Lewis acid sites and NH_4^+ interacting with Brønsted acid sites (Si-OH-Al), respectively [2]. The aged samples exhibited intense NH_3 absorption peaks on Lewis acid and Brønsted acid sites, indicating the well-maintained Cu^{2+} ions and structure, consistent with the EPR (Fig. 5) and XRD (Fig. 2) results.

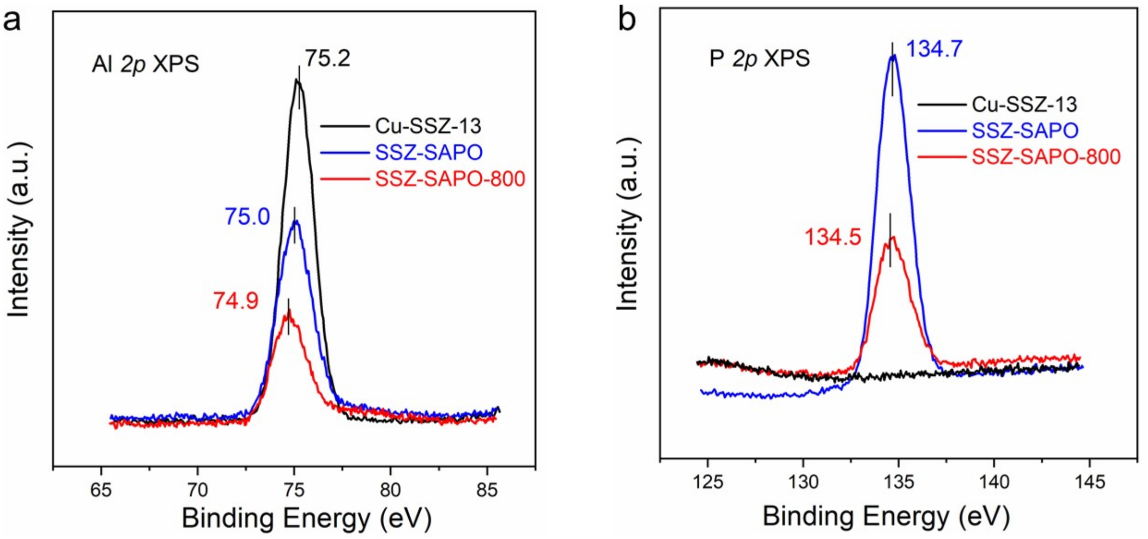

Fig. S5. Al 2p and P 2p XPS spectra of samples

The Cu-SSZ-13 and SSZ-SAPO samples showed the Al 2p XPS peaks at 75.2 and 75.0 eV, respectively. Clearly, the addition of H-SAPO-34 in the composite shifted the Al 2p XPS peak to lower angle, which is probably attributed to the presence of P-Al bond. The SSZ-SAPO-800 sample presented the Al 2p XPS peak at 74.9 eV, lower than that in SSZ-SAPO. This indicates that the Al in SSZ-13 might interact with P upon aging at 800 °C. The P 2p XPS results (Fig. S5b) support this hypothesis, which displayed that the XPS peak in SSZ-SAPO shifted from 134.7 to 13.5 eV after aging at 800 °C.

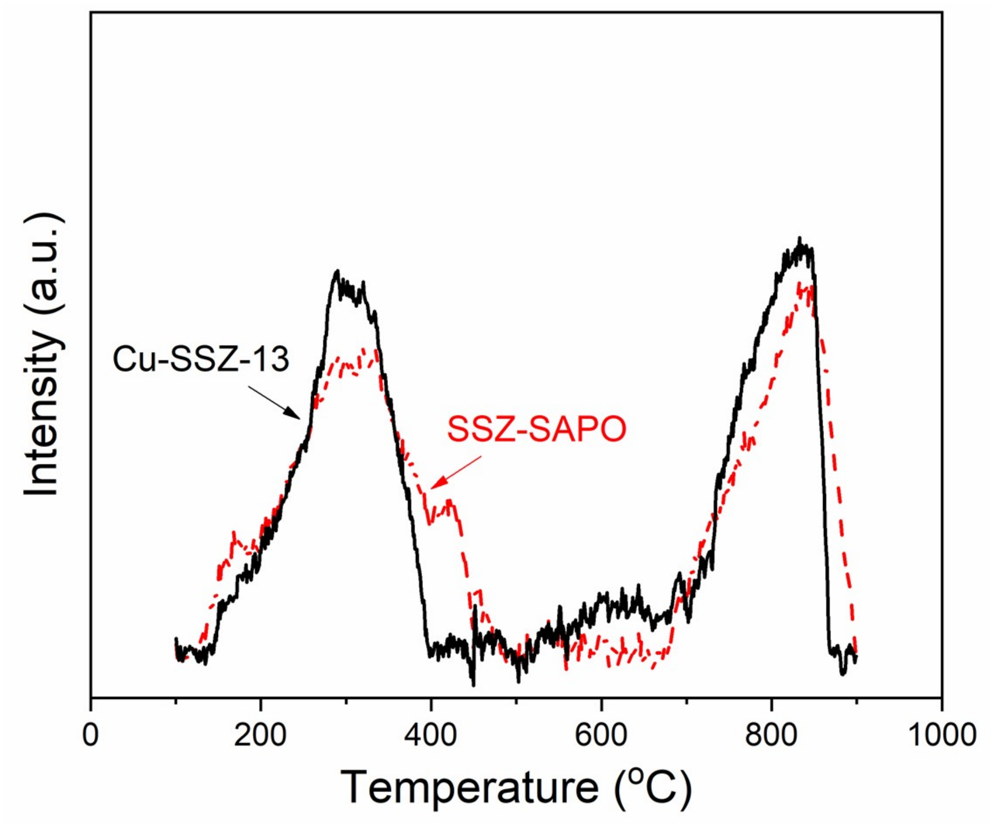

Fig. S6. H_2-TPR profiles of Cu-SSZ-13 and SSZ-SAPO samples. The curve of SSZ-SAPO was copied from Fig. 7.

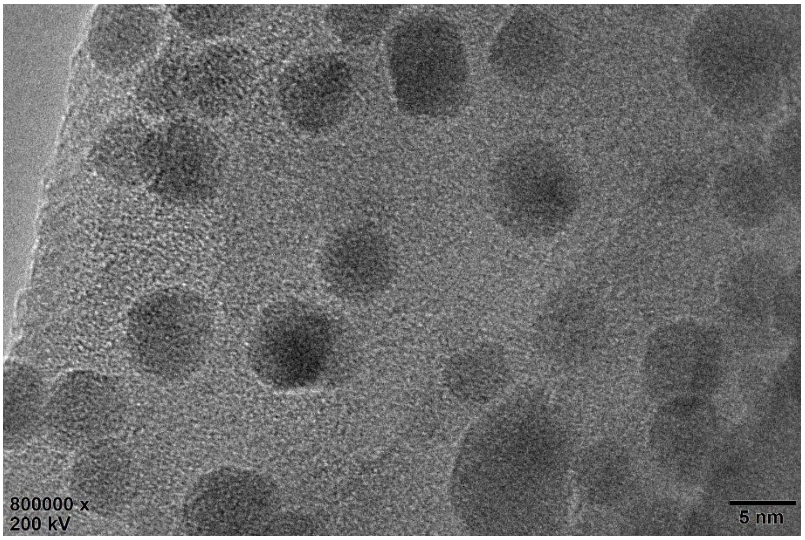

Fig. S7. The TEM image of SSZ-SAPO-750.

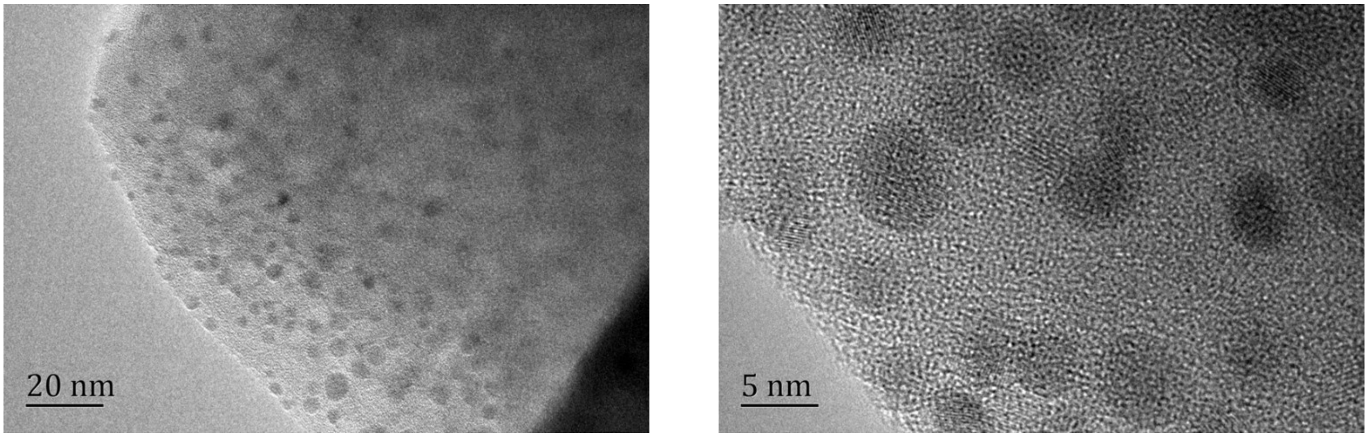

Fig. S8. The TEM image of SSZ-SAPO-850.

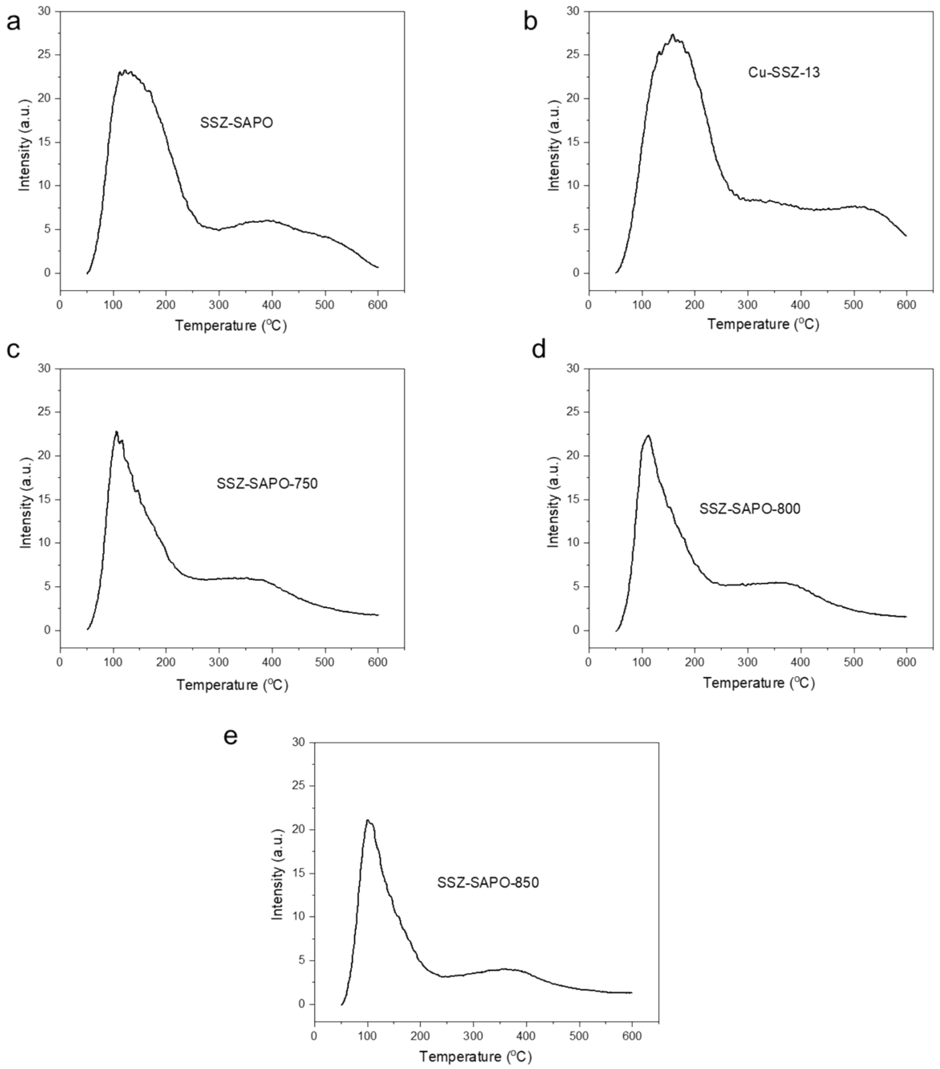

Fig. S9. NH_3-TPD results of the samples.

After HTA, the NH_3 desorption peak in 400-500 °C decreased in the composite samples, indicating the decrease of Brønsted sites.

References

[1] L. Xie, F. Liu, X. Shi, F.-S. Xiao, H. He, Appl. Catal. B: Environ. 179 (2015) 206-212.

[2] Y. Shan, J. Du, Y. Yu, W. Shan, X. Shi, H. He, Appl. Catal. B: Environ. 266 (2020) 118655.
